# Enhancing T cell persistence of CAR-redirected T cells in solid tumors

**DOI:** 10.1186/2051-1426-2-S3-P244

**Published:** 2014-11-06

**Authors:** Sonia Guedan, Shannon E McGettigan, Avery D Posey, Jihyun Lee, Omkar Kawalekar, Prachi R Patel, Brian Keith, Carl June

**Affiliations:** 1 University of Pennsylvania, Philadelphia, PA, USA; 2 University of Pennsylvania, Hatboro, PA, USA; 3 University of Pennsylvania, Poland

## 

T cell persistence is likely to promote long-term anti-tumor effects after adoptive T cell transfer. We have recently shown that incorporation of the ICOS intracellular domain into chimeric antigen receptors (CARs) significantly increased Th17 cell persistence *in vivo*, compared to CARs with CD28 or 4-1BB intracellular domains [1]. Here, we hypothesized that CD4^+ ^and CD8^+ ^T cells require distinct cytokine and costimulation signals for optimal persistence. To test this hypothesis, we compared the *in vivo *antitumor effects and persistence of combined CD4^+ ^T cells (bulk or Th17-polarized) and CD8^+ ^T cells redirected with CARs containing CD28, 4-1BB or ICOS-based costimulatory domains. Using multiple mouse tumor models, we demonstrate that the ICOS intracellular domain significantly enhanced the *in vivo *persistence of CAR-expressing CD4^+ ^T cells, and that both persistence and tumor infiltration were further enhanced by culturing these cells under Th17-polarizing conditions. Importantly, Th17-polarized CD4^+ ^T cells expressing an ICOS-based CAR significantly increased the circulatory persistence of bulk CD8^+ ^T cells expressing either CD28- or 4-1BB-based CARs. We further demonstrate that the antitumor effect of CAR-expressing CD8^+ ^T cells was enhanced when co-injected with ICOS-redirected Th17 cells. Collectively, our data suggest that combining Th17 CD4^+ ^T cells redirected with an ICOS-based CAR with CD8^+ ^CAR-T cells will enhance their persistence and antitumor efficacy.
